# Diagnostic Features of Common Oral Ulcerative Lesions: An Updated Decision Tree

**DOI:** 10.1155/2016/7278925

**Published:** 2016-10-03

**Authors:** Hamed Mortazavi, Yaser Safi, Maryam Baharvand, Somayeh Rahmani

**Affiliations:** ^1^Department of Oral Medicine, School of Dentistry, Shahid Beheshti University of Medical Sciences, Tehran, Iran; ^2^Department of Oral and Maxillofacial Radiology, School of Dentistry, Shahid Beheshti University of Medical Sciences, Tehran, Iran

## Abstract

Diagnosis of oral ulcerative lesions might be quite challenging. This narrative review article aims to introduce an updated decision tree for diagnosing oral ulcerative lesions on the basis of their diagnostic features. Various general search engines and specialized databases including PubMed, PubMed Central, Medline Plus, EBSCO, Science Direct, Scopus, Embase, and authenticated textbooks were used to find relevant topics by means of MeSH keywords such as “oral ulcer,” “stomatitis,” and “mouth diseases.” Thereafter, English-language articles published since 1983 to 2015 in both medical and dental journals including reviews, meta-analyses, original papers, and case reports were appraised. Upon compilation of the relevant data, oral ulcerative lesions were categorized into three major groups: acute, chronic, and recurrent ulcers and into five subgroups: solitary acute, multiple acute, solitary chronic, multiple chronic, and solitary/multiple recurrent, based on the number and duration of lesions. In total, 29 entities were organized in the form of a decision tree in order to help clinicians establish a logical diagnosis by stepwise progression.

## 1. Introduction

Ulcerations are characterized by defects in the epithelium, underlying connective tissue, or both. Due to diversity of causative factors and presenting features, diagnosis of oral ulcerative lesions might be quite challenging [1–4]. This narrative review paper, however, focuses on the duration and the number of lesions in order to build a diagnostic decision tree.

For the purpose of this article, if an ulcerative lesion lasts for two weeks or longer, it is considered chronic; otherwise, it is regarded as an acute ulcer [1, 2]. Recurrent ulcers, on the other hand, present with a history of similar episodes with intermittent healing [3]. The term* solitary *indicates the presence of a single ulcerative lesion whereas the term* multiple *describes the presence of several ulcerative lesions [5]. In order to arrive at a definitive diagnosis, it is imperative to consider differential diagnoses. This is the cognitive process of integrating logic and knowledge into a series of stepwise decisions. All lesions that cannot be excluded initially should be included in the differential diagnosis, followed by laboratory tests and additional investigations to narrow the diagnosis. According to the literature, many cases of oral malignant ulcerations were misdiagnosed as nonneoplastic lesions up to several months before the definite diagnosis was established [6–8]. Valente et al. reported a case of squamous cell carcinoma misdiagnosed as a denture-related traumatic ulcer [6]. Meanwhile, de Sant' Ana dos Santos et al. reported misdiagnosis of lip SCC as actinic cheilitis [7]. A case of gingival SCC masquerading as an aphthous ulcer was also reported by Kumari et al. [8]. This time elapse might jeopardize patients' overall prognosis; therefore, attempts should be done to come to timely diagnosis via more logical routes such as decision trees rather than test-and-error methods. A decision tree is a flowchart that organizes features of lesions so that the clinician can make a series of orderly decisions to reach a logical conclusion. To use the decision tree, the clinician begins from the left side of the tree, makes the first decision, and proceeds to the far right of the tree, where the names of entities are listed [9, 10]. This narrative review article aims to introduce an updated decision tree for diagnosing oral ulcerative lesions on the basis of their diagnostic features.

## 2. Search Strategy

General search engines and specialized databases including PubMed, PubMed Central, Medline Plus, EBSCO, Science Direct, Scopus, Embase, and authenticated textbooks were used by the first author and the corresponding author to find relevant topics by means of MeSH keywords such as “oral ulcer,” “stomatitis,” and “mouth diseases.” Related English-language articles published since 1983 to 2015 in both medical and dental journals including reviews, meta-analyses, original papers (randomized or nonrandomized clinical trials, prospective or retrospective cohort studies), case reports, and case series on oral ulcers, stomatitis, and oral disease were appraised.

## 3. Results

**Figure 1:**
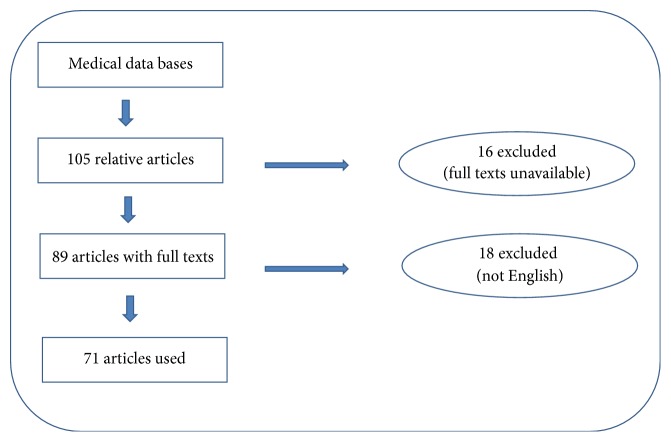
Flow chart for choosing eligible articles.

Out of a total of 105 relative articles, 34 were excluded due to lack of full texts or being written in languages other than English. Finally, 4 textbooks and 71 papers were selected including 32 reviews, 27 case reports or case series, and 12 original articles (Figure 1).

**Figure 2:**
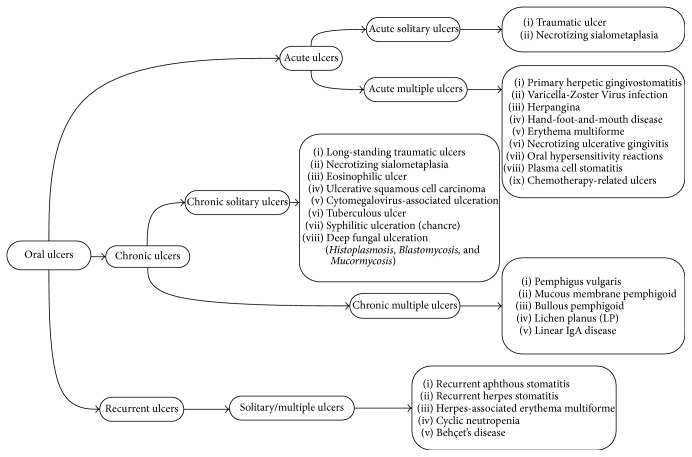
Decision tree of oral ulcerative lesions.

In this narrative review article, oral ulcerative lesions were categorized into three major groups: acute, chronic, and recurrent ulcers (Tables 1 [Table tab2]–3) and into five subgroups: solitary acute, multiple acute, solitary chronic, multiple chronic, and solitary/multiple recurrent, based on the number and duration of lesions. In total, 29 entities were organized in the form of a decision tree (Figure 2) in order to help clinicians establish a logical diagnosis by stepwise progression. The first decision to be made is whether the ulcerative lesion is of an acute, chronic, or recurrent nature; thereafter, the lesion(s) should be placed in one of the five subgroups. Then, the clinician can consult the list of diagnoses in the relevant category.

### 3.1. Clinical Features of Oral Ulcerative Lesions

#### 3.1.1. Acute Solitary Ulcers

**Figure 3:**
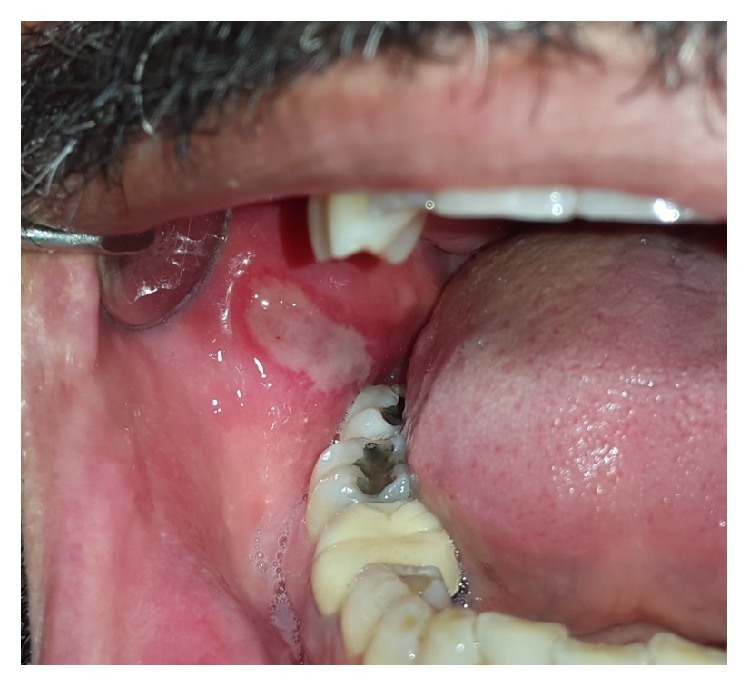
Traumatic ulcer coated with pseudomembrane and surrounded by inflammatory halo.

*Traumatic Ulcer*. Traumatic injuries of the oral mucosa are quite common. They are caused by mechanical damage (contact with sharp foodstuff; accidental biting during mastication, talking, or even sleeping) and thermal, electrical, or chemical burns [11]. Traumatic ulcers are most common on the tongue, lips, and buccal mucosa [5]. According to Chen et al., traumatic lesions of the oral cavity were mostly seen on the buccal mucosa (42%), followed by the tongue (25%) and the lower lip (9%) [12]. Noteworthy, traumatic ulcers are more common in men than women (male : female ratio of 2.7 : 1) [12]. These lesions may persist for a few days or even several weeks, especially in the case of tongue ulcers due to repeated insults to the tissues [5, 33]. The borders of traumatic ulcers are usually slightly raised and reddish, with a yellowish-white necrotic pseudomembrane that can be readily wiped off (Figure 3). Ulcers on the lip vermilion usually have a crusted surface [5]. Traumatic ulcers normally become painless within three days after the injury had been eliminated and, in most cases, heal within 10 days [5].

*Necrotizing Sialometaplasia*. Necrotizing sialometaplasia (NS) is a self-limiting, benign, inflammatory disease of the salivary glands more frequently seen in middle-aged men. Although the main etiology is not clear; many authors believe that local infarction due to ischemia of the salivary tissue is the causative factor. Meanwhile, a number of potential predisposing factors have been suggested such as sharp direct local trauma (local anesthesia, intubation, and surgical procedures), use of ill-fitting dentures, violent or provoked vomiting (in patients with bulimia), upper respiratory infection, and radiotherapy [11, 34]. NS appears as a crater-like ulcer with indurated and well-delineated borders [34]. More than 75% of all cases occur on the posterior part of the palate, followed by the lower lip, retromolar pad, sublingual region, tongue, and larynx [11, 34]. The size of the lesion ranges from less than 1 cm to over 5 cm [11]. The healing time of NS varies as well. Chen et al. showed a considerable improvement in healing process after 10 days [12]. However, complete healing is usually observed only after five to seven weeks [11, 34].

#### 3.1.2. Acute Multiple Ulcers

*Primary Herpetic Gingivostomatitis*. Primary herpetic gingivostomatitis is the most common pattern of symptomatic* herpes simplex virus* (HSV) infection. Over 90% of cases are caused by HSV type 1, and the remainder are caused by HSV2 [12]. It can be asymptomatic or very mild in young patients but is associated with more severe general symptoms in the elderly [1]. Most cases occur between the ages of six months and 5 years, with peak prevalence between 2 and 3 years [11]. Fever, nausea, anorexia, and irritability are initial symptoms. Oral manifestations consist of a generalized gingivitis followed, after 2-3 days, by pin-headed vesicles that readily rupture and give rise to painful ulcers covered by a yellowish pseudomembrane. They often coalesce into larger ulcers. Keratinized and nonkeratinized mucosa can be affected, and the number of the lesions is quite variable [13]. In many cases, punched-out erosions along the free gingival margin have been reported [11]. Submandibular lymphadenitis, halitosis, and difficulty in swallowing are noted in most cases [13, 35]. Noteworthy, some adult patients may present with pharyngotonsillitis. In addition, involvement of the oral mucosa anterior to Waldeyer's ring is encountered in roughly 10% of patients [11]. The ulcers usually heal spontaneously after 5 to 7 days, with no scarring, but may persist for two weeks in severe cases [13, 35, 36].

**Figure 4:**
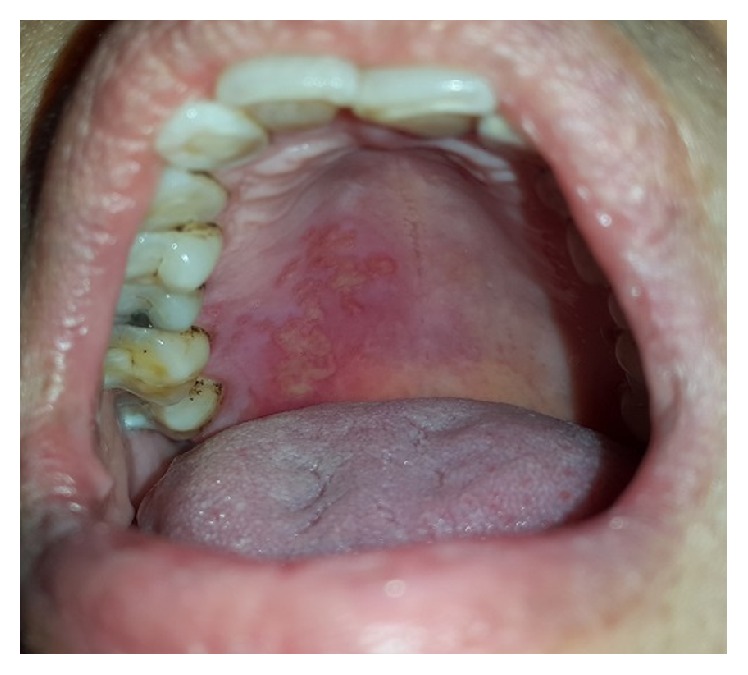
Herpes zoster presenting as small and coalesced ulcers with scalloped borders, unilateral and zosteriform pattern.

*Herpes Zoster Infection (Shingles)*. Herpes zoster infection (HZI) is a less common viral infection brought about by the reactivation of* Varicella Zoster Virus* (VZV) [14, 37, 38], which may happen spontaneously or as a result of immune system deficiency. Increased age, trauma (from dental procedures), psychological stress, malignancy, radiotherapy, and immunocompromised conditions such as organ transplantation, immunosuppressive therapy, and HIV infection are contributory factors for VZV reactivation. The incidence of HZI is 1.5–3 cases per 1,000 persons, which increases to 10 per 1,000 in people over 75 years of age [15, 37]. Majority of HZI involve the thoracic and lumbar dermatomes, but nearly 13% of patients present with involvement of the trigeminal nerve branch, most commonly the ophthalmic branch. The condition is acutely painful and patients with involvement of the maxillary branch experience a prodromal phase of unilateral pain, burning, and tenderness, usually on the palate (Figure 4). After several days, painful, clustered ulcers of 1–5 mm in diameter appear on the hard palate or buccal gingivae in a characteristic unilateral pattern. The ulcers tend to heal within 10–14 days. Development of blisters and ulcers on the mandibular gingivae and tongue is indicative of mandibular branch involvement. This entity is self-limiting, and management of oral lesions is directed toward pain control, supportive care, and hydration. Use of acyclovir, valacyclovir, or famciclovir is also effective in treating HZI when started within 72 hours of disease onset [3, 13].

**Figure 5:**
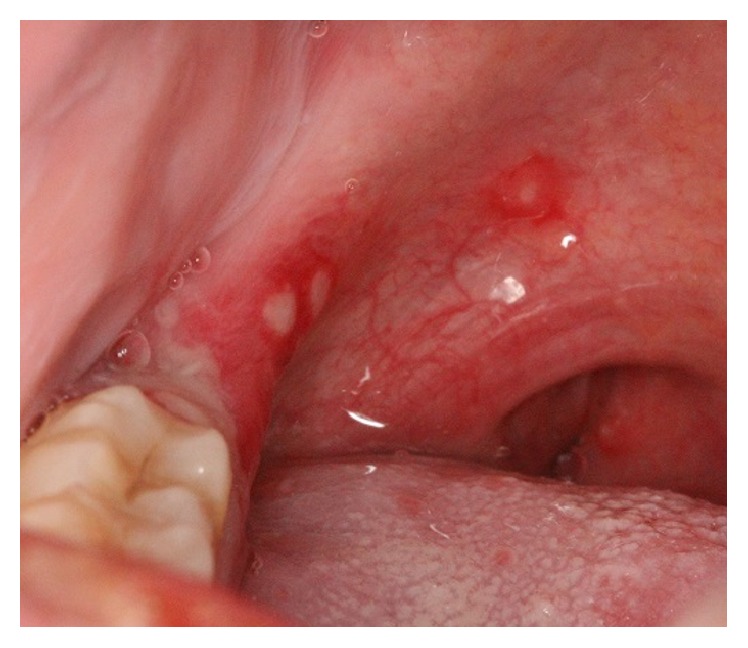
Small and symmetrical ulcers of herpangina on the soft palate and retromolar pad.

*Herpangina*. Herpangina presents as multiple vesicular exanthema and ulcers of the oropharynx, soft palate, and tonsillar pillars [16, 17] (Figure 5). Children under 10 years of age are usually affected, and outbreaks occur in epidemics in summer.* Coxsackie virus* A genotypes 1–10 (CAV1–10), A12, A16, and A22 and* Coxsackie virus* B genotypes (CBV2–5) and* Echo virus* 18 and* Entero virus* 71 identified as etiologic factors [16]. It is a self-limiting disease and management directed toward control of oral pain and fever. Effective antiviral medications for* Coxsackie virus* infections are not available yet [17].

*Hand-Foot-and-Mouth Disease*. Hand-foot-and-mouth disease (HFM) is one of the common causes of morbidity among children below 10 years of age [16, 39]. All of the patients have skin rash, especially on the hands and feet and 30% on the buttocks. Oral ulcers are usually located on the tongue, hard and soft palate, and buccal mucosa. Management is similar to herpangina [3].

*Erythema Multiforme*. Erythema multiforme (EM) is a mucocutaneous hypersensitivity reaction with different etiologies. It is characterized by irregular red macules, papules, and vesicles that coalesce with each other to grow larger and make plaques on the skin called target lesions [40]. Oral lesions usually appear as erythematous macules on the lips and buccal mucosa, followed by bullae and ulcerations with irregular borders and inflammatory halo. Bloody encrustations can be observed on the lips, which is a diagnostic feature [18, 41]. EM can be triggered by medications such as sulfonamide, penicillin, cephalosporins, quinolones, analgesics, and nonsteroidal anti-inflammatory drugs (NSAIDs) or several infections (*herpes simplex virus*,* Epstein-barr Virus*,* Cytomegalovirus*,* Varicella Zoster Virus*, fungal agents, and parasites) [40]. EM typically affects young adults (20–40 years) and teenagers, but the onset might be as late as 50 years of age or elder [41]. There is a male predilection with male to female ratio of 3 : 2 [42]. According to recent evidence, EM has been categorized as minor, major, Steven-Johnson Syndrome or Toxic Epidermal Necrolysis. The former is the mildest type and the latter is the most severe one [40]. Prodromal signs such as fever, lymphadenopathy, headache, malaise, cough, and sore throat may be noticed one week prior to onset of mucocutaneous erythema or blisters [18, 40, 41]. Treatment mainly depends on the severity of clinical presentations. In the mild forms, healing takes place within 10 to 20 days; therefore, patients only need local wound care, liquid diet, and topical analgesics or anesthetics for pain control [40, 41].

**Figure 6:**
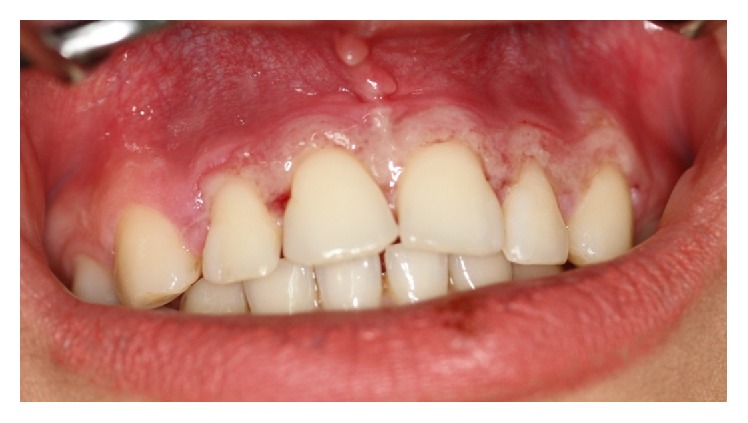
Punched-out necrotic ulcers of NUG on the gingival papillae.

*Necrotizing Ulcerative Gingivitis*. Necrotizing ulcerative gingivitis (NUG) is an acute infectious disease of the gingivae. It is characterized by “punched-out” ulcerations, and necrosis on the papillary and marginal gingivae (Figure 6) as well as severe gingival pain and bleeding [19, 43]. There are some predisposing factors such as smoking, poor oral hygiene, preexisting gingivitis, malnutrition, psychological stress, and HIV infection. The above-mentioned factors usually lead to immunodysregulation including depressed polymorphonuclear leukocytes, antibody response, and lymphocyte mitogenesis [43]. NUG usually affects young adults (18–20 years of age), and it is estimated to be seen in 0.5% to 11% of the population [19, 43]. The diagnosis of NUG is based on three essential symptoms: sore gums, bleeding gums, and, the most diagnostic criterion, ulceration and necrosis of the interdental papillae [19]. Treatment is based on mechanical removal of tartar with local (chlorhexidine, 0.12% twice daily) and systemic (amoxicillin 250 mg and metronidazole 250 mg, three times a day for 7 days) delivery of antimicrobial agents. Adequate treatment usually prevents progression of the lesions. Healing is expected in a few days, whereas inadequate treatment can lead to deterioration of lesions in the form of necrotizing ulcerative periodontitis (NUP) [19, 43].

*Oral Hypersensitivity Reactions*. Oral hypersensitivity reactions (OHRs) have a variety of manifestations: acute onset of EM ulcers, red and white reticular lesions such as lichenoid reactions, fixed drug eruption (usually seen as ulcers on the lip vermilion after exposure to drugs with resolution on withdrawal and relapse on rechallenge), swelling of the lips, and oral allergy syndrome (itching with or without swelling of oral structures and oropharynx) [3, 44, 45].

*Plasma Cell Stomatitis*. Plasma cell stomatitis (PCS) was first described in the late 1960s and early 1970s as a hypersensitivity reaction and likely a contact stomatitis to a component of chewing gum. This entity usually occurs few days after exposure and presents as erythematous macular areas of oral cavity. Ulceration, epithelial sloughing, and desquamation may also be seen. Gingivae is the mostly affected site. Angular cheilitis with fissuring and dry atrophic lips have been found in patients with PCS [3]. OHRs and PCS are usually self-limiting. Nevertheless, pain control and anti-inflammatory agents can help diminish the healing time [3, 44, 45].

*Chemotherapy-Related Ulcers*. Chemotherapeutic agents may cause ulcers through direct or indirect mechanisms. Bone marrow suppression and immune response of oral mucosa, which leads to bacterial, fungal, or viral infections, happen during indirect effect of chemotherapeutic agents. Other medications cause oral ulcerative lesions via direct impact on replication and growth of the oral epithelial cells [3, 46]. Kolbinson et al. demonstrated that early changes in the oral mucosa such as erythema and ulceration appear between 5 and 7 days after onset of chemotherapy [47]. It is also noted that these lesions are considered as risk factors for systemic infections [48]. Moreover, Dreizen et al. pointed out that 30% to 50% of patients undergoing chemotherapy develop oral lesions [49]. After completion of chemotherapy, the lesions resolve spontaneously; however, anti-inflammatory drugs may be useful in minimizing chemotherapy-related ulcers [3, 46].

#### 3.1.3. Chronic Solitary Ulcers

*Sustained Traumatic Ulcers*. Chronic injuries of oral mucosa may lead to solitary long standing ulcerative lesions; therefore, traumatic ulcer can also be classified as a chronic solitary ulcer. This entity has been reported by Pattison as a self-inflicted gingival lesion in patients who were seeking prescriptions for narcotic drugs [50]. Chronic traumatic ulcerations usually occur on the tongue, lips, and buccal mucosa [11] as ulcerative areas surrounding a central removable, yellow fibrinopurulent membrane. In many cases, the lesion develops a raised, rolled border of hyperkeratosis immediately adjacent to the area of ulceration [11, 51, 52]. Most traumatic ulcers become painless and heal within 10 days. However, some lesions persist for several weeks because of continued traumatic insults, irritation by the oral liquids, or secondary infection. There are different treatment modalities, but coating the ulcerated surface with fluocinonide or triamcinolone acetonide in an emollient base after meals and before bed time usually relieves pain and decreases duration of healing [5].

*Necrotizing Sialometaplasia*. Although this lesion usually occurs on the palate, it can be seen anywhere from oral mucosa, which contains salivary glands including the retromolar trigon and the lips. NS initially presents as a tender erythematous nodule, followed by a deep ulcer with a yellowish base [3]. Average age of patients is 46 years and it is more common in males. It resembles squamous cell carcinoma and ulcerated mucoepidermoid carcinoma to a large extent during its ulcerated phase [5]. NS is mainly a self-limiting lesion, and healing time may be varied from 2 to 12 weeks according to the severity of the lesion [5, 11, 34]. Therefore, NS can be classified as an acute or chronic solitary ulcer.

**Figure 7:**
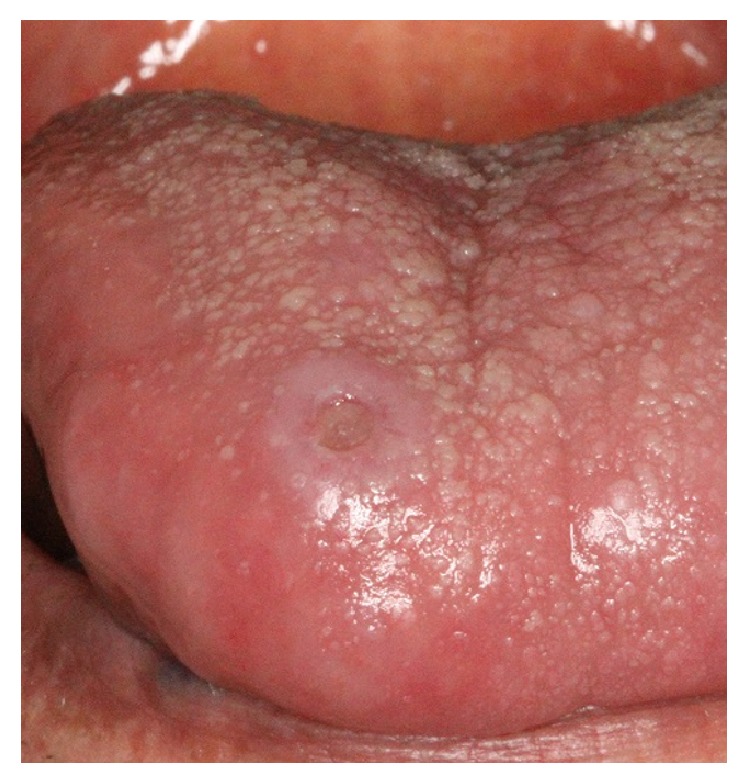
Punched-out eosinophilic ulcer on dorsal surface of the tongue with a raised keratotic border.

*Eosinophilic Ulcer*. Eosinophilic ulcer (EU) or traumatic ulcerative granuloma with stromal eosinophilia (TUGSE) is a chronic solitary ulcer of oral mucosa, which is most frequently seen in patients aged 40−60 years [21], but occurs in young and elderly patients as well [53]. Male to female ratio is 1 : 1, or slightly more prevalent in women [53]. The most frequently affected site is the tongue (about 60% of cases), followed by buccal mucosa, retromolar region, floor of the mouth, and lips [3, 21]. Eosinophilic ulcer manifests as a slow-healing ulcer with a rolled or elevated border mimicking a squamous cell carcinoma (Figure 7). They ranged from 0.5 to several cm in size. In two-thirds of cases, the lesion may be asymptomatic and persists for months. The main etiology is not clear, but trauma has been elicited in 20% to 50% of cases [21]. The duration of healing time ranges from 1 week to 1 year [21]. Surgical excision is the accepted treatment method, and recurrence is rare. In addition, intralesional corticosteroids, oral corticosteroids, topical antibiotics, and cryotherapy have been also suggested [53].

**Figure 8:**
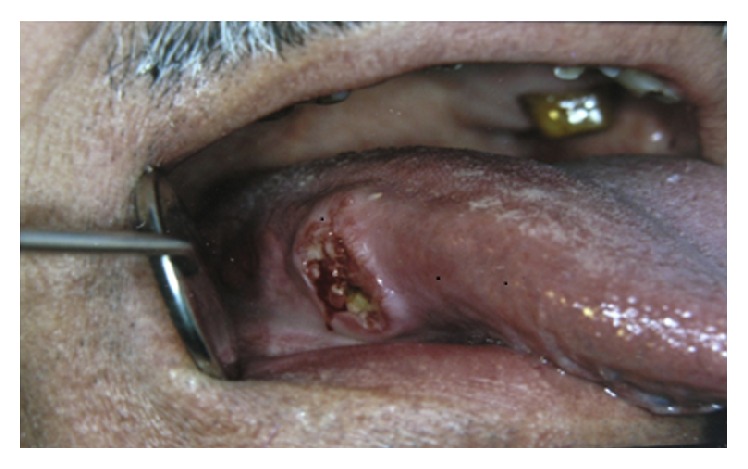
Ulcerative SCC of the tongue with rolled borders.

*Ulcerative Squamous Cell Carcinoma*. Squamous cell carcinoma (SCC) represents about 95% of all oral malignancies [22, 54]. It presents as a red, white, red-white, exophytic, or ulcerative lesion. SCC is a persistent ulcer in the oral cavity, which is of high importance especially on the lips. SCC is often asymptomatic; therefore, patients usually are not aware of it until it has become relatively progressive. The classic ulcerative SCC is described as a craterlike lesion having a rolled, indurated border and a velvety base (Figure 8). It may be covered with a crust when occurring on the vermilion [5, 22]. The mostly affected sites in the oral cavity are lower lip, floor of the mouth, and ventral and lateral borders of the tongue. Lesions are usually solitary, but in rare cases multifocal [5]. According to Wood and Goaz, a lesion is most likely a SCC if the patient is male, older than 40 years, smokes or drinks heavily, no evidence of trauma or systemic disease exists, serologic findings are negative, and the lesion is not located on the posterolateral region of the hard palate [5]. Ulcerative form of SCC is locally destructive; thereby timely and correct diagnosis of oral SCC plays a key role in the improvement of patients prognosis and survival rate [3, 55]. There is no single treatment for oral SCC; however, various therapeutic modalities from surgery, radiotherapy, and chemotherapy to combination different methods have been introduced [54].

*Cytomegalovirus-Associated Ulceration*. Cytomegalovirus (CMV) is a member of herpes virus group. About 80% of adults have serologic evidence of CMV infection without clinical outcome. Cytomegalovirus-related ulcers occur more commonly under conditions of significant immunodeficiency, especially severe HIV disease [3, 55]. Oral ulcers are usually single, painful, large, and necrotic, with minimally rolled border, which affects keratinized and nonkeratinized mucosa. On rare occasions, multiple ulcerations are seen [3, 20]. CMV ulcers might last for several weeks or months. Treatment can be accomplished by valganciclovir, ganciclovir, or cidofovir. Topical anesthesia or systemic analgesics might be useful for pain relief. Good hydration and dietary modification should also be considered [3].

*Tuberculous Ulcer*. Some granulomatous diseases such as tuberculosis and leprosy can cause ulcerative lesions in the oral cavity [23, 56, 57]. The World Health Organization (WHO) has estimated 9.4 million incident cases and 11.1 million prevalent cases of TB globally [23, 57]. Tuberculosis rarely affects oral mucosa, roughly 1.4% of all TB cases with a male to female ratio of 4 : 1 [23]. The classic oral lesion presents as a solitary ulcer usually with an undermined edge most commonly on the tongue, followed by gingivae, floor of the mouth, palate, lips, and buccal mucosa. Meanwhile, it may be ragged and indurated and is often painful [24, 25]. The differential diagnosis of tuberculous ulcer includes traumatic ulcer, syphilitic ulcer, and oral SCC [24].

*Syphilitic Ulceration (Chancre)*. Primary syphilitic ulceration usually occurs as a result of orogenital or oroanal contact with an infectious lesion. It rarely affects the mouth and often remains undiagnosed because of its temporary duration. Almost one to three weeks after acquisition, a chancre develops as a solitary ulcer usually on the lips or rarely on the tongue, pharynx, or tonsils. The upper lip is more commonly affected in males and the lower lip in females, probably due to the anatomy involved with fellatio and cunnilingus. The ulceration is usually deep, with a red purple or brown base ragged rolled border, and usually an accompanying cervical lymphadenopathy. Traumatic ulceration and squamous cell carcinoma might be quite similar to syphilitic chancre. Detailed history of sexual and social life style helps approach the diagnosis of primary syphilis [26, 58, 59].

*Deep Fungal Ulceration (Histoplasmosis, Blastomycosis, and Mucormycosis)*. Oral mucosal lesion in histoplasmosis is usually secondary to pulmonary involvement and occurs in a significant percentage of patients with disseminated histoplasmosis. It begins as an area of erythema that converts to a granulomatous ulcer.

The most frequent feature of oral blastomycosis is a nonspecific, painless, verrucous ulcer with indurated borders, often mistaken for oral SCC.

The most common oral sign of mucormycosis is ulceration of the palate, which results from necrosis due to invasion of a palatal vessel. Lesion has also been observed on the gingivae, lips, and alveolar ridge [3].

#### 3.1.4. Multiple Chronic Ulcers

*Vesiculobullous Diseases*. Some vesiculobullous diseases such as pemphigus vulgaris (PV), mucous membrane pemphigoid (MMP), and bullous pemphigoid (BP) present as multiple and chronic oral ulcerative lesions [3].

Pemphigus vulgaris (PV) is a chronic vesiculobullous mucocutaneous autoimmune disease characterized by loss of cell adhesion (acantholysis) and blister formation [60]. About 90% of patients with PV develop oral lesions, and in more than 50% of cases they are the first sign of disease. Oral lesions begin as bullae on noninflamed base. More frequently, clinicians notice shallow irregular ulcers, because the bullae rapidly rupture. The edge of lesions continues to extend over a period of weeks until they involve large areas of oral cavity. The lesions usually start on the buccal mucosa; however, palate and gingivae are other commonly affected sites. Gingival involvement might be in the form of desquamative gingivitis, which is a characteristic feature for PV [3]. Oral lesions appear several months predating skin lesions [3, 27]. Final diagnosis often takes more than 5 months from the onset of disease. Meanwhile, coexisting candidiasis may mask the typical features of PV lesions. There is a small group of PV patients whose disease remains confined to the oral cavity. These patients often have negative results on direct and indirect immunofluorescence testing [3]. Early diagnosis is an important aspect of patient management when lower doses of medication can be used for shorter periods of time to control the disease. The mainstay of treatment remains high doses of systemic corticosteroids, usually given in dosages of 1 to 2 mg/kg/d [3, 60].

Mucous membrane pemphigoid (MMP) has been known by different names including benign mucous membrane pemphigoid, cicatricial (scarring) pemphigoid, and ocular cicatricial pemphigoid [61]. MMP is a common immune-mediated subepithelial blistering disease mainly affecting oral mucosa (over 90%); however, skin lesions are also present in 20% to 30% of cases. The most affected site in the oral cavity is gingivae followed by buccal mucosa and palate. It occurs twice as frequently in females and is generally seen in patients more than 50 years old [3, 5, 62, 63]. Desquamative gingivitis is the most common presentation of the disease, which can be the only feature of MMP. Blood blisters which result from bleeding into bullae are a diagnostic feature of MMP in the oral cavity. Use of topical or systemic corticosteroids is considered as an acceptable treatment for MMP. Furthermore, when there is ocular involvement dapsone therapy is recommended [3].

Bullous pemphigoid (BP) is the most common subepithelial blistering disease, which occurs chiefly in patients over the age of 60 [3]. In this entity, oral mucosal involvement is not common. According to Budimir et al., oral mucosal lesions are found in only 16.6% of cases [64], which are similar to PV but are smaller and less painful. Meanwhile, extensive labial involvement which is common in PV is not present in BP. Desquamative gingivitis has been mentioned as the most frequent oral manifestation in BP and gingivae may be the only affected site [3]. Clinically, oral lesions are not distinguishable from PV or MMP, but early remission of BP is more common [64, 65]. Noteworthy, BP has been reported in conjunction with other diseases such as multiple sclerosis, malignancies, or medications particularly diuretics [3]. Bullous pemphigoid is self-limiting and may last from a few months to 5 years. Topical clobetasol or betamethasone has been suggested in the management of localized oral lesions, whereas, in more extensive disease, use of systemic corticosteroids alone or in combination with immunosuppressive drugs is recommended [3, 64].

**Figure 9:**
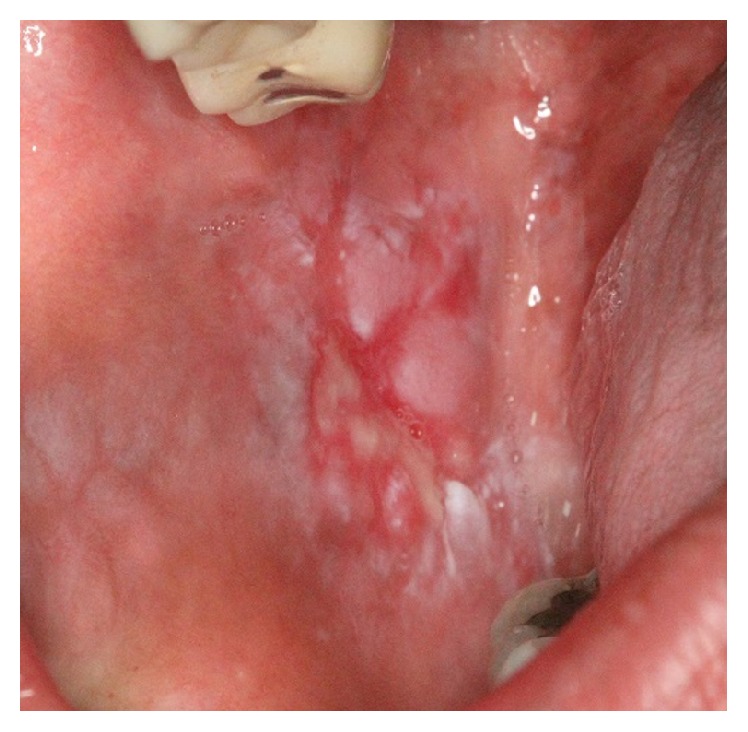
Ulcerative lichen planus on the buccal mucosa presenting as a central ulcer with pseudomembrane and keratotic plaques at the periphery.

*Lichen Planus (LP)*. Lichen planus (LP) is a chronic, autoimmune, mucocutaneous disease. Most LP patients are middle-aged, and it is rare in children. The disease occurs more commonly in women than in men with a female/male ratio of approximately 2 : 1. The reported prevalence of LP is up to 5%, while the prevalence of oral LP (OLP) is 0.1% to 2.2% [28, 66]. OLP may occur alone or in combination with skin or other mucosal involvement [28]. There are different subtypes of OLP with different clinical presentations: reticular (lace-like keratotic mucosal configuration, Wickham's striae), atrophic (keratotic changes combined with mucosal erythema), erosive/ulcerative (pseudomembrane covered ulcerations combined with keratosis and erythema), bullous (vesiculobullous presentation combined with reticular or erosive patterns), and popular/plaque like (keratotic changes with elevation adjacent to the normal mucosa) (Figure 9). Reticular, popular, and plaque-like lesions are generally asymptomatic whereas bullous, erosive and ulcerative forms are generally associated with pain. Atrophic or erosive LP involving the gingivae results in desquamative gingivitis. This condition has been also found in MMP and PV [3]. The most frequently affected site in the oral cavity is buccal mucosa, followed by the tongue, gingivae, palate, and vermilion border [66]. OLP is considered a premalignant lesion. The risk of malignancy has been estimated to be 0.4%–3.7%, which often develops after 10 years. Hence, OLP should be followed up for a long time. Topical or systemic corticosteroids are usually recommended in management of OLP [66].

*Linear IgA Disease*. Linear IgA disease (LAD) or linear IgA dermatitis is an autoimmune subepithelial mucocutaneous disease. LAD affects both adults and children. Peak of incidence is between sixth and seventh decades of life in adult patients, with a twofold predilection for females. The pediatric variant is called chronic bullous dermatitis of childhood or juvenile dermatitis herpetiform [67]. Oral involvement in LAD has been estimated to be between 5% and 70% in the form of vesicles, painful ulcerations or erosions, and erosive gingivitis/cheilitis. The most common affected site in the oral cavity is hard and soft palate, followed by tonsillar pillars, buccal mucosa, tongue, and gingivae [68]. In a case report by Chan et al., oral lesions were the only presentation of LAD for 5 years [69]. Oral lesions are usually managed with the use of topical steroids, but dapsone therapy for more severe cases is recommended. Resistant cases may require systemic corticosteroids [3].

#### 3.1.5. Recurrent Ulcers (Solitary/Multiple)

**Figure 10:**
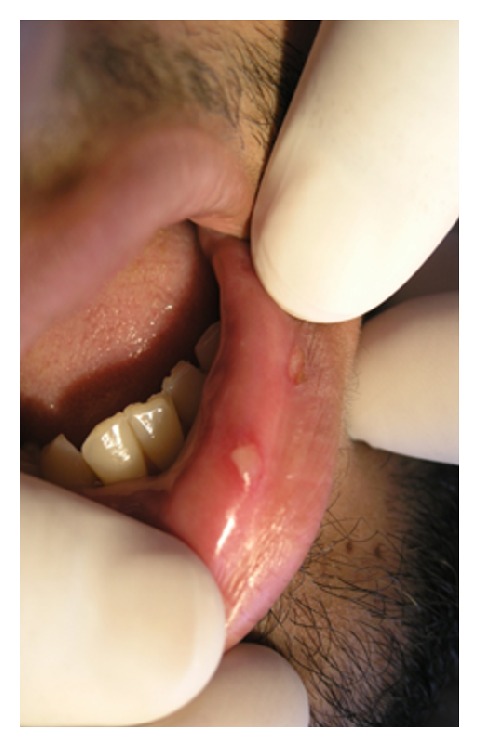
Recurrent aphthous stomatitis as a symmetrical ulcer with pseudomembrane and inflammatory halo.

*Recurrent Aphthous Stomatitis*. Recurrent aphthous stomatitis (RAS) is the most common inflammatory disease of the oral mucosa with a global prevalence of 0.5% to 75% and female predilection [70]. The first episode of RAS most frequently commences in the second decade of life. The lesions usually begin with prodromal burning sensation 2 to 48 hours before an ulcer appears [3]. Oral aphthous ulcers typically occur as painful, symmetrically round fibrin-covered mucosal defects with an erythematous border and most commonly on nonkeratinized mucosa in healthy patients (Figure 10). However, it can be seen on the keratinized mucosa especially in patients with immune deficiency. Three clinical types of RAS have been identified: minor-type (Mikulicz) is smaller than 1 cm in diameter (usually 2-3 mm) and heals spontaneously in two weeks. This type constitutes 80–90% of all aphthous ulcers. Major-type (Sutton ulcer) is usually 1–3 cm in size, and lasts for 10 days to 6 weeks or even longer. More than 60% of Sutton ulcers heal with scarring. Major type accounts for about 10% of RAS. Herpetiform aphthae appears as very small (1-2 mm), extremely painful, and numerous ulcers (up to 100 lesions). About 32% of lesions heal with scarring [70, 71]. Diagnosis is based on patient's history and pattern of ulcers. Laboratory evaluation is mandatory when (a) episodes of lesions become more severe, (b) lesions begin after the age of 25, and (c) general symptoms are accompanied by lesions [3]. RAS is self-limiting, but in severe cases topical or systemic corticosteroids are recommended [70, 71].

**Figure 11:**
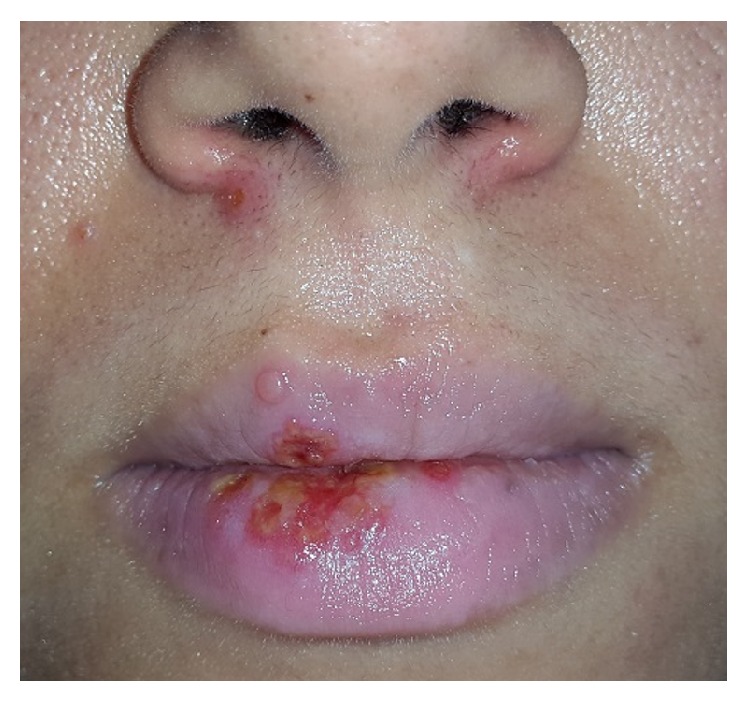
Recurrent herpes labialis presented as multiple vesicles and scalloped-border erosions coated with crust.

*Recurrent Herpes Stomatitis*. Herpes simplex virus can establish latency in the trigeminal ganglia and periodically reactivate to cause recurrent herpetic stomatitis (RHS). There are two subgroups: recurrent herpes simplex labialis (HSL) (Figure 11), which is more commonly seen in healthy subjects. It begins as vesicles that rupture soon, which leave superficial crusted ulcers and heal without scarring. Recurrent intraoral herpes (RIH) is more common in immunocompromised patients. However, RIH in immune competent patients is limited to the keratinized mucosa, especially on the hard palate usually as clustered and unilateral vesicles. Common triggers of RHS are physical/emotional stress, UV light, cold weather, hormonal changes, upper respiratory tract illness, and lip/mouth trauma [3, 72]. Although the lesions are self-limiting, symptomatic treatment by using ice or lanolin is recommended. Applying acyclovir ointment 5% every 2 hours since the prodromal phase until the lesions subside has been suggested as well. Elective dental treatments should be deferred in patients with active lesions to prevent aerosolization of the virus [71].

*Herpes-Associated Erythema Multiform*. Erythema multiform (EM) can be induced by several infectious agents, in particular HSV [73, 74]. According to Ng et al., HSV DNA has been detected in 50% of patients with recurrent idiopathic EM [74] typically affecting young adults (20–40 years) and is more common in men with the ratio of 3 : 2 [73]. It can be found several days or weeks following an episode of HSV, and the lips are mostly affected [73]. The diagnosis is based on clinical features and is more straightforward when target lesions with preceding or coexisting HSV infection present. History of recurrence has been found in 25% of all EM cases. Patients usually experience 2 to 24 episodes in a year. The duration of disease ranges from 2 to 36 years (mean of 10 years). Acyclovir (200 mg, 5 times a day, for 5 days) is recommended for management of lesions [73].

*Cyclic Neutropenia*. Cyclic neutropenia (CN) is an immunodeficiency syndrome, characterized by regular periodic oscillations in the circulating neutrophil count from normal to neutropenic levels every 21 days, and lasting for 3–6 days. Patients with cyclic neutropenia are usually asymptomatic but during neutropenic episodes suffer from fever of unknown origin, gingivitis, stomatitis, aphthous-like ulceration, cellulitis, perirectal abscess, and severe systemic pyogenic infections [75]. The severity and recurrence of oral ulcers in CN are similar to those of ulcers in major RAS; additionally, periodontal destruction in CN is as severe as what is seen in aggressive periodontitis [29].

*Behçet's Disease*. Behçet's disease (BD) is a systemic immune-mediated vasculitis characterized by the presence of recurrent oral and genital ulcers, ocular inflammation, and skin lesions. The etiology and pathogenesis of BD are unknown. BD can affect any age groups, but onset before puberty and after the sixth decade of life is relatively rare. The most common age of presentation is around the third decade of life, with a balanced male/female ratio. Recurrent and painful oral ulcers are present in 90% to 100% of patients with BD. The diagnosis of BD is based on clinical criteria. Presence of recurrent oral aphthous-like ulcers (minor, major, or herpetiform ulcers, which recur at least three times within a period of 12 months) along with two of the following: genital ulcers, ocular lesions (anterior uveitis, posterior uveitis, vitreous cellularity, or retinal vasculitis), and skin lesions (erythema nodosum, pseudofolliculitis or papulopustular lesions, or acneiform papulae in postadolescent patients without any steroid treatment, and positive pathergy test) establishes the diagnosis [30–32]. Glucocorticoids, colchicine, azathioprine, cyclosporine, tacrolimus, anti-TNF-alpha (infliximab and etanercept), thalidomide, and rituximab are all recommended for treatment. Topical corticosteroids are not optimally effective against oral lesions. Treatment with infliximab causes complete resolution of recurrent oral ulcers [30].

## 4. Discussion

Oral ulcerative lesions are categorized into solitary acute, multiple acute, solitary chronic, multiple chronic, and recurrent lesions.

Acute oral ulcerations result from traumatic insults, viral or bacterial infections, allergy, or cancer chemotherapy. Acute traumatic ulcers usually present as solitary lesions of nonspecific clinical shapes (Figure 3) [11, 12], whereas viral stomatitis appear as multiple small symmetrical ulcers, which sometimes coalesce to form larger lesions with scalloped borders (Figure 4) [11, 13]. Allergic stomatitis tend to involve any site intraorally with various clinical features usually with a history of synchronous exposure to contactants or systemic allergens [3, 44]. Although chemotherapy-induced stomatitis can be quite diffuse, its commencement is chronologically related to therapeutic agents, which differentiate it from other acute oral ulcerations [46, 47]. Some bacterial stomatitis present as either acute multiple punched- out necrotic ulcerations (e.g., NUG and NUP) (Figure 6) in patients with malnutrition or immunocompromised state [19, 43], or single chronic ulceration of which tuberculous ulcer presents as an undermined lesion [24]. When confronting a single chronic ulcer, it would appear to be prudent to preclude oral squamous cell carcinoma (Figure 8) first due to its poor prognosis and grave morbidity [5, 22]. In addition, in the category of solitary chronic ulcers, history of lung disease or dealing with animals propose histoplasmosis or blastomycosis, while presence of debilitating disease such as diabetes mellitus suggest mucormycosis [3]. On the other hand, multiple map-like or asymmetrical ulcerations suggest the presence of mucocutaneous vesiculobullous diseases; among them, pemphigus is of high potency for extension, which might lead to death [3, 27]. Despite chronic lesions with ongoing progression or sustained clinical features a history of repeated resolution and relapse prompts the clinician to rank recurrent oral ulcerations higher in the differential diagnosis. Recurrent aphthous stomatitis (Figure 10) and recurrent herpes are two common entities in this category with the former being more frequent in the nonkeratinized mucosa and the latter in the keratinized mucosa [3, 27].

## 5. Conclusion

The newly updated decision tree includes 29 oral ulcerative lesions based on duration and number of lesions, which helps clinicians establish a stepwise method to rule out improbable conditions to arrive at a logical diagnosis.

## Figures and Tables

**Table 1:** Clinical characteristics of acute oral ulceration [2, 3, 11–20].

Lesion	Gender predominance	Age predilection	Location	Shape of ulcer	Number of ulcers	Distinguishing features
Traumatic ulcer	Men	NA^*∗*^	Tongue, lip, buccal mucosa	Symmetrical/asymmetrical	Solitary	Slightly raised and reddish borders, necrotic pseudomembrane, which heals within 10 days

Necrotizing sialometaplasia	Men	Middle age	Posterior palate, lower lip, retromolar pad	Crater-like	Solitary	Ulcers with indurated borders, self-limiting after 5 to 7 weeks

Primary herpetic gingivostomatitis	NA	2-3 years	Keratinized and nonkeratinized mucosa	Ulcers with scalloped borders and erythematous halo	Multiple	Prodromal fever, nausea, anorexia, and irritability generalized gingivitis, painful ulcers covered by a yellowish pseudomembrane, submandibular lymphadenitis, halitosis, and dysphagia, self-limiting after 5 to 7 days

Herpes zoster infection (shingles)	NA	>50 years of age	Hard palate, gingivae, tongue	Ulcers with scalloped borders, Zosteriform pattern	Multiple	Prodromal unilateral pain, clustered small ulcers with characteristic unilateral pattern, self-limiting, healing within 10–14 days

Herpangina	NA	<10 years of age	Oropharynx, soft palate, tonsillar pillars	Small ulcers	Multiple	Vesicular exanthema and ulcers

Hand-foot-and-mouth disease	NA	<10 years of age	Tongue, hard, and soft palate, buccal mucosa	Small ulcers	Multiple	Oral ulcers along with skin rash on the hands and feet

Erythema multiforme	men	20–40 years	Lips, buccal mucosa, tongue	Large and confluent	Multiple	Prodromal skin target lesions, bullae and ulcerations with irregular borders and inflammatory halo, bloody encrustations on the lips

Necrotizing ulcerative gingivitis	NA	Young adults (18–20 years of age)	Papillary and marginal gingivae	Crater like	Multiple	Sore gums, bleeding gums, ulceration, and necrosis of the interdental papillae, fetid odor, fever, and malaise

Oral hypersensitivity reactions	NA	NA	Any site intraorally	Several clinical manifestations	Multiple	Lichenoid reactions, fixed drug eruption, swelling of the lips, and oral allergy syndrome

Plasma cell stomatitis	NA	NA	Gingivae, sulcus, buccal mucosa	Desquamative gingivitis	Multiple	Ulceration, epithelial sloughing, and desquamation, angular cheilitis, atrophic fissured lips, self-limiting

Chemotherapy-related ulcers	NA	NA	Any site intraorally	Asymmetrical ulcers	Multiple	Erythema and ulceration 5 to 7 days after onset of chemotherapy, spontaneously resolution after completion of chemotherapy

^*∗*^NA: not assigned.

**Table 2 tab2:** Clinical characteristics of chronic oral ulceration [1–4, 11, 20–28].

Lesion	Gender predominance	Age predilection	Location	Shape of ulcer	Number of ulcers	Distinguishing features
Sustained traumatic ulcer	NA^*∗*^	NA	Tongue, lips, buccal mucosa	Symmetrical/asymmetrical	Solitary	Central removable, yellow fibrinopurulent membrane; a raised, rolled border of hyperkeratosis immediately adjacent to the area of ulceration

Necrotizing sialometaplasia	Men	Average age: 46 years	Palate, retromolar pad, lips	Craterlike	Solitary	Deep ulcer with a yellowish base, self-limiting, healing time between 2 and 12 weeks

Eosinophilic ulcer	Women	40–60 years	Tongue, buccal mucosa	Punched out	Solitary	Slow-healing ulcer with a rolled border, surrounding erythema or keratosis, mostly asymptomatic, healing time between 1 week and 1 year

Ulcerative squamous cell carcinoma	Men	>40 years	Lower lip, floor of the mouth, tongue	Craterlike	Solitary	Rolled, indurated borders and a velvety base

Cytomegalovirus-associated ulceration	NA	NA	Keratinized and nonkeratinized mucosa	Large ulcer	Solitary, rarely multiple	Painful, necrotic, with minimally rolled border, mostly in immunocompromised patients

Tuberculous ulcer	Men	NA	Tongue, gingivae, floor of the mouth	Undermined borders	Solitary	May be ragged and indurated, often painful

Syphilitic ulceration (chancre)	NA	NA	Lips, tongue, palate	Clean-based ulceration	Solitary	Red purple or brown base, ragged rolled border, cervical lymphadenopathy

Oral blastomycosis	Men	NA	Any oral mucosal surface	Nonspecific ulcer	Solitary	Painless, irregular rolled borders and verrucous mucosal hyperplasia, painful, concomitant pulmonary lesions on chest radiographs

Oral mucormycosis	NA	NA	Palate	Verrucous ulcer	Solitary	Large deep necrotic ulcer, associated with concomitant pulmonary disease and an underlying debilitating disease

Pemphigus vulgaris	NA	NA	Buccal mucosa, palate, gingivae	Map-like	Multiple	Shallow irregular ulcers with peripheral extension, positive Nikolsky sign, desquamative gingivitis

Mucous membrane pemphigoid	Women	>50 years	Gingivae, buccal mucosa, palate	Nonspecific-appearing erythema and erosions	Multiple	Desquamative gingivitis as the most common oral presentation, blood blisters, skin lesions in 30–40% of cases

Bullous pemphigoid	NA	>60 years of age	Gingivae	Discrete vesicle formation	Multiple	Desquamative gingivitis as the most frequent oral manifestation, self-limiting, early remission

Lichen planus	Women	Middle age	Buccal mucosa, tongue, gingivae		Multiple	Accompanying reticular or papular lesions, desquamative gingivitis, painful

Linear IgA disease	Women	6th and 7th decades of life	Hard and soft palate, tonsillar pillars, buccal mucosa	Clinically indistinguishable from the oral lesions of MMP	Multiple	Vesicles, painful ulcerations or erosions, desquamative gingivitis/cheilitis

^*∗*^NA: not assigned.

**Table 3:** Clinical characteristics of recurrent oral ulceration [2–4, 11, 20, 29–32].

Lesion	Gender predominance	Age predilection	Location	Shape of ulcer	Number of ulcers	Distinguishing features
Recurrent aphthous stomatitis	Women	Beginning at 2nd decade	Nonkeratinized mucosa in healthy patients	Symmetrical	Solitary/multiple	Prodromal burning, painful, round fibrin covered with erythematous borders, 3 clinical types: minor, major, herpetiform, self-limiting

Recurrent herpetic stomatitis	NA^*∗*^	NA	Keratinized mucosa especially hard palate in healthy patients	Scalloped borders	Multiple	Two subgroups: recurrent herpes simplex labialis, recurrent intraoral herpes, unilateral

Herpes-associated erythema multiform	Men	20–40 years	Lips	Map-like	Multiple	Bullae and ulcerations with irregular borders and inflammatory halo, bloody encrustations on the lips

Cyclic neutropenia	NA	Begins in childhood	Any oral mucosa exposed to trauma	Aphthous-like	Multiple	Episodic ulcers with erythematous halo, concomitant fever, periodontitis, marked gingival recession, and systemic infections

Behçet's disease	Balanced male/female ratio	Between the ages of 25 and 40	Anywhere on the oral or pharyngeal mucosa	Aphthous-like	Multiple	Concomitant genital ulcers, ocular inflammation, and skin lesions

^*∗*^NA: not assigned.
